# Free‐Breathing Hepatic Oxygen Extraction Fraction (OEF) Mapping Using Radial GESSE

**DOI:** 10.1002/mrm.70550

**Published:** 2026-08-06

**Authors:** Ke Zhang, Simon M. F. Triphan, Felix T. Kurz, Congcong Fu, Na Zhang, Christian H. Ziener, Mark E. Ladd, Heinz‐Peter Schlemmer, Hans‐Ulrich Kauczor, Oliver Sedlaczek

**Affiliations:** ^1^ Department of Diagnostic and Interventional Radiology Heidelberg University Hospital Heidelberg Germany; ^2^ Translational Lung Research Center (TLRC) German Center for Lung Research (DZL) Heidelberg Germany; ^3^ Department of Diagnostic and Interventional Radiology With Nuclear Medicine Thoraxklinik at Heidelberg University Hospital Heidelberg Germany; ^4^ Divison of Radiology German Cancer Research Center Heidelberg Germany; ^5^ Division of Neuroradiology Geneva University Hospitals Geneva Switzerland; ^6^ Paul C. Lauterbur Research Center for Biomedical Imaging Shenzhen Institute of Advanced Technology, Chinese Academy of Sciences Shenzhen China; ^7^ Division of Medical Physics in Radiology German Cancer Research Center Heidelberg Germany; ^8^ Faculty of Physics and Astronomy Heidelberg University Heidelberg Germany; ^9^ Faculty of Medicine Heidelberg University Heidelberg Germany

**Keywords:** artificial neural network, oxygen extraction fraction, quantitative BOLD, radial gradient‐echo sampling of spin‐echo

## Abstract

**Purpose:**

Noninvasive assessment of hepatic oxygenation is relevant for evaluating liver both in acute and long‐term liver pathologies. However, existing methods are limited to single‐slice acquisitions and require breath‐holding. This study aims to develop and evaluate a motion‐robust technique for whole‐liver oxygen extraction fraction (OEF) mapping using a radial gradient‐echo sampling of spin‐echo (rGESSE) sequence in combination with an artificial neural network (ANN) for quantification.

**Methods:**

Seven healthy volunteers were scanned using a 1.5 T MRI system with an 18‐channel body coil. A multi‐slice rGESSE sequence with radial sampling was employed under free‐breathing conditions to acquire volumetric liver data. Signal processing included bias‐field correction and 3D median filtering. OEF, deoxygenated blood volume (DBV), and transverse relaxation rate (*R*_2_) were estimated using a trained feedforward ANN based on simulated qBOLD signal models.

**Results:**

Whole‐liver OEF maps were successfully obtained in all volunteers under free‐breathing. Representative parameter maps showed consistent spatial patterns and anatomical correspondence. The mean hepatic OEF across subjects was 55.75% ± 6.88%, and the mean DBV was 0.568 ± 0.039. Comparison with literature values suggested a systematic overestimation, likely arising from a combination of model approximations, residual B_0_ inhomogeneity, motion effects, and acquisition‐specific sensitivities. ANN‐based fitting outperformed standard least‐squares regression in terms of stability and artifact suppression.

**Conclusion:**

This study demonstrates the feasibility of using rGESSE combined with ANN analysis for free‐breathing, whole‐liver OEF mapping. The proposed approach allows for noninvasive, volumetric hepatic oxygenation assessment with improved motion robustness, offering potential for clinical application in liver disease diagnosis and monitoring.

## 1 Introduction

Significant alterations in regional hepatic oxygen delivery and consumption are recognized as key markers of acute and subacute hepatic cellular dysfunction. Impaired oxygenation is associated with a range of pathological processes, ranging from acute ischemic liver damage to chronic oxidative stress, which is thought to contribute to the progression of various liver diseases. The conditions include complications after liver transplantation or traumatic hemorrhagic shock while the subacute ones are related to common diseases such as alcohol‐ and obesity‐related liver injury, and thus the development and progression of hepatocellular carcinoma (HCC) [1, 2, 3]. Accurate assessment of hepatic oxygen metabolism could therefore play a crucial role in early diagnosis, risk stratification, and monitoring of disease progression or response to therapy [4, 5, 6].

The gold standard for measuring oxygen extraction fraction (OEF) in vivo has traditionally been positron emission tomography (PET) using ^15^O‐labeled radiotracers [7, 8, 9]. However, the use of ^15^O‐PET is limited in clinical settings due to its short radiotracer half‐life, the need for an on‐site cyclotron, and the exposure of patients to ionizing radiation. These limitations have spurred interest in developing noninvasive, radiation‐free alternatives that can be integrated into routine clinical imaging protocols.

Quantitative BOLD (qBOLD) MRI is one such technique that enables the separation of contributions to the BOLD signal from venous blood oxygenation (Y_v_) and deoxygenated blood volume (DBV) [10, 11, 12]. By isolating these physiological parameters, qBOLD allows for regional and absolute in vivo measurement of blood oxygen saturation. Originally developed and validated in the brain, qBOLD has been successfully applied in human brain studies [10, 12] and in preclinical animal models [11], demonstrating its potential for assessing tissue oxygenation in a variety of clinical scenarios.

To improve the efficiency and precision of OEF measurement, Gradient‐Echo Sampling of the Spin Echo (GESSE) has been introduced as an alternative acquisition strategy [12]. GESSE pulse sequence is a sequence that allows a hybrid mapping of T_2_ and T_2_* relaxation times; it was developed to separate macroscopic and microscopic magnetic field inhomogeneities in MRI in microstructural brain imaging [13]. In GESSE, a set of gradient echoes (GREs) are embedded around the spin echo (SE) of a single SE sequence [10]. This sequence structure allows a simultaneous acquisition of a set of images corresponding to different GRE times (TEs) and therefore allows simultaneous T_2_ and T_2_* mapping. The measurement of T_2_ and T_2_* using GESSE was developed and applied for OEF mapping [10, 14, 15, 16]. Furthermore, GESSE is insensitive to RF pulse errors and does not suffer from significant field distortions, therefore providing a robust mapping technique in OEF imaging.

Recent efforts have extended MRI‐based OEF mapping to the liver [17]. One approach demonstrated the feasibility of estimating hepatic Y_v_ using a multi‐echo gradient‐echo (multi‐GRE) sequence acquired in a single breath‐hold [18]. While promising, breath‐hold acquisitions inherently limit spatial coverage and may not be suitable for patients with impaired respiratory function. Moreover, liver motion during free breathing remains a major technical challenge for quantitative imaging.

In this study, we investigate the feasibility of free‐breathing hepatic OEF mapping using a respiratory motion‐robust radial GESSE (rGESSE) sequence. This method enables multi‐slice coverage and enhanced motion insensitivity due to the radial sampling trajectory. To analyze the rGESSE signal, we employed a feedforward artificial neural network (ANN), which allows for fast, computation‐efficient and accurate estimation of OEF while accounting for complex signal dependencies [14, 16]. Our aim was to demonstrate that rGESSE‐based OEF mapping, combined with ANN fitting, offers a practical and reliable approach for noninvasive, whole‐liver assessment of oxygen extraction under free‐breathing conditions.

## 2 Methods

### 2.1 Subjects and Data Acquisition

Seven healthy volunteers (5 female, 2 male, aged 22–40 years) were examined prospectively using an 18‐channel body and spine receive RF coil on a 1.5 T scanner (Aera, Siemens Healthineers AG, Erlangen, Germany). All participants provided written informed consent, and the study was approved by the institutional ethics committee.

For radial sampling, evenly distributed radial angles were used to allow for online reconstruction provided by the vendor. Specific sequence parameters were as follows: FOV = 400 × 400 mm^2^, radial projections/slice = 160, radial ordering scheme = linear, interleaved slice ordering, matrix size = 200 × 200 × 20, resolution = 2 × 2 × 3 mm^3^, slice gap = 0.9 mm, TE = 43 ms, TR = 100 ms, echo spacing ΔTE ≈ 1 ms, total number of echoes = 64, number of echoes before echo center = 16, flip angle = 90°, bandwidth = 1000 Hz/PX, acquisition time was about 5 min 18 s. The rGESSE sequence diagram using radial sampling is illustrated in Figure 1. The asymmetry of the gradient echoes around the spin echo in our rGESSE acquisition was chosen to optimize sensitivity to hepatic T_2_, which at 1.5 T is relatively short (~40–60 ms). Placing more echoes before the spin echo would increase the effective TE of the central echo and reduce T_2_ weighting in liver tissue, decreasing sensitivity to relevant signal decay for DBV and OEF estimation. The rGESSE acquisition was performed under free‐breathing conditions. Projections were acquired in sequential radial order, providing near‐uniform k‐space coverage over time and allowing for vendor‐based online reconstruction. Slices were acquired in an interleaved order to reduce crosstalk. While through‐plane motion may still affect individual voxels, the combination of radial sampling and ANN‐based inversion was expected to provide stable and robust OEF and DBV estimates.

**FIGURE 1:**
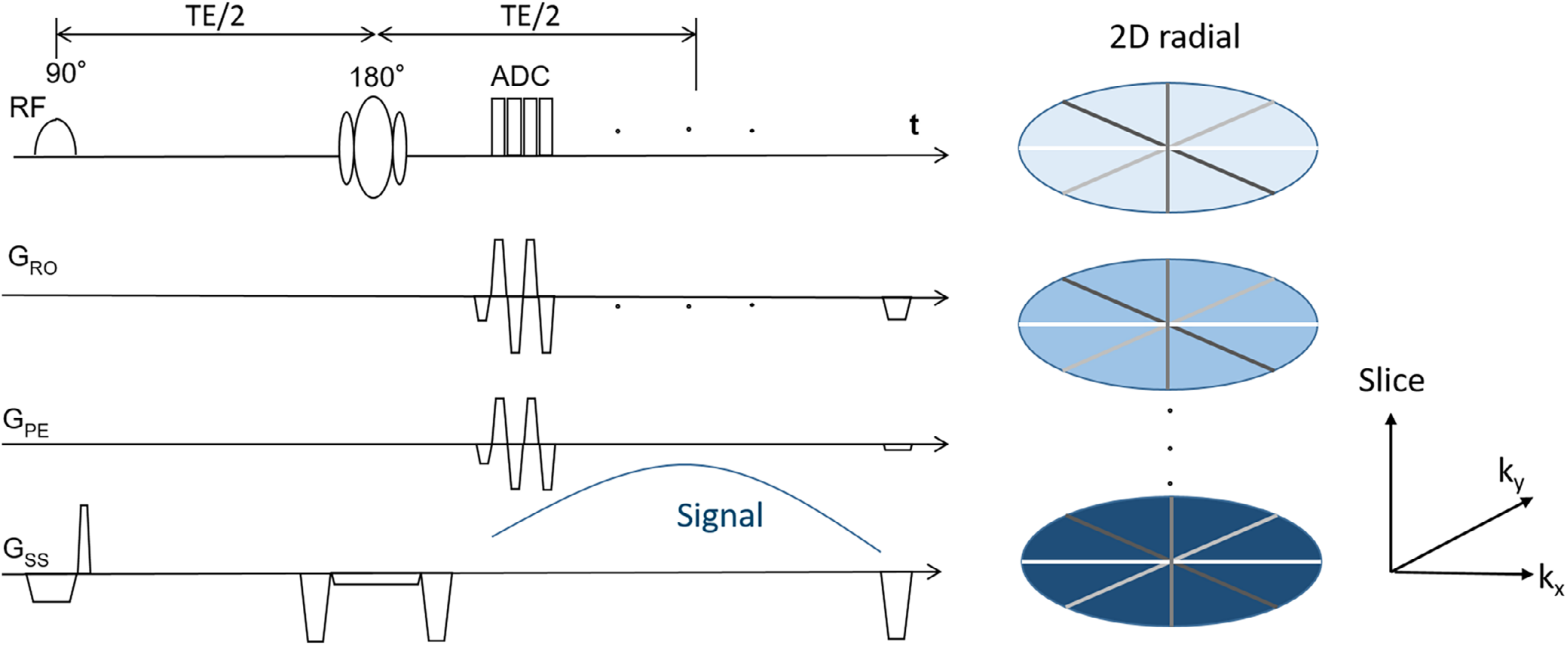
Diagram of radial GESSE (rGESSE) sequence. The rGESSE acquisition consists of a slice‐selective 90° excitation pulse followed by a refocusing pulse generating a single spin echo at TE. Gradient echoes are sampled both before and after the spin echo in an asymmetric arrangement. The readout samples 64 gradient echoes per TR, allowing simultaneous estimation of *R*_2_ and *R*_2_′. Radial k‐space sampling is performed in a sequential radial order, providing uniform coverage. Slice interleaving is used to minimize crosstalk, but no respiratory gating or breath‐holding is applied, enabling free‐breathing acquisition.

### 2.2 Preprocessing and Signal Correction

Before quantitative analysis, bias field correction was applied to the center echo images to correct for receiving field inhomogeneities in signal intensity. This bias correction map was then propagated across all echoes to ensure consistency throughout the time series. To enhance the signal‐to‐noise ratio (SNR) and suppress residual imaging artifacts, a 2D median filter was applied to each slice of the full echo dataset, as previously described [18]. For each voxel, the output value was computed as the median of a 3 × 3 neighborhood within the slice centered on the corresponding voxel in the input image. This step was chosen to maintain sensitivity to physiologically relevant heterogeneity in DBV and OEF. While the median filter alters the noise distribution, it provides a practical balance between noise suppression and preservation of voxel‐level signal characteristics. Linear low‐pass filtering was not applied, as it may blur small‐scale variations and reduce sensitivity to local physiological contrasts.

### 2.3 OEF Estimation Using Artificial Neural Network

To estimate the oxygen extraction fraction (OEF), we implemented a feedforward ANN trained to perform nonlinear regression of rGESSE signal dynamics. The ANN was chosen over traditional least‐squares regression (LSR) due to its improved ability to handle noisy, nonlinear signal behavior and its superior computational efficiency, which is particularly advantageous for multi‐slice, high‐resolution data. The ANN was implemented in MATLAB and consisted of a 64‐dimensional input layer, two hidden layers (32 and 10‐dimensional), and a 4‐dimensional output layer (Figure S1). The ANN was trained based on the full qBOLD model [10] under the static dephasing regime. The characteristic function assumes static dephasing around cylindrical vessels, similar to prior brain studies. Artificial BOLD signals with a known ground truth were simulated for plausible ranges of the input variables SSE, *R*_2_, *λ*, and OEF [14]. These variables are: static spin‐echo signal (SSE), spin–spin relaxation rate (*R*_2_), the vascular parameter (*λ*) or DBV, and OEF: To reduce overfitting artifacts, noise was added to the simulated BOLD signals before the actual ANN training was initialized. The ANN was trained using simulated qBOLD signal evolutions spanning physiologically relevant parameter ranges. Training, validation, and testing were performed using independent simulated datasets to avoid data leakage. The ANN was not explicitly trained on motion‐corrupted signals; robustness to respiratory motion was instead achieved through the use of a free‐breathing radial acquisition and reconstruction strategy, which yields temporally stable signal evolutions for ANN inference.

### 2.4 Statistical Analysis

In all subjects, 20 × 20 voxel regions of interest (ROIs) were drawn on the parametric maps at anatomically matching locations within the liver, kidney, spleen, and skeletal muscle. Liver ROIs were placed in the right lobe, avoiding large vessels and bile ducts; kidney ROIs in the renal cortex; spleen ROIs in homogeneous parenchyma, avoiding organ edges; and skeletal muscle ROIs were placed in the paraspinal muscles, avoiding fascia and visible vessels. All ROIs were manually delineated by a single experienced operator and reviewed for consistency across subjects to ensure reproducibility. The mean, standard deviation, and median of the values within each ROI were calculated for all tissues. Statistical analyses were performed in MATLAB. Pairwise comparisons between tissues were conducted using the Wilcoxon signed‐rank test. For all tests, the level of significance was defined at *p* < 0.05. Unless otherwise stated, all results are presented as mean values ± standard deviation.

We additionally acquired data using a single‐slice 2D GRE (32 echoes) sequence and a 3D GRE *B*_0_ mapping sequence during free‐breathing for comparison. The 2D GRE data were analyzed following the methods described in Wengler et al. [18].

## 3 Results

Representative rGESSE images and corresponding signal time courses from different ROIs in the liver, spleen, and kidneys are presented in Figure 2. The temporal signal evolution across the 64 echoes demonstrates clear organ‐specific contrast behavior, which was consistent across subjects. Quantitative fitting of these signals using both conventional LSR and the proposed ANN approach is also shown. Compared to LSR, ANN fitting provided smoother and more physiologically plausible curves, particularly in low‐SNR regions such as muscle.

**FIGURE 2:**
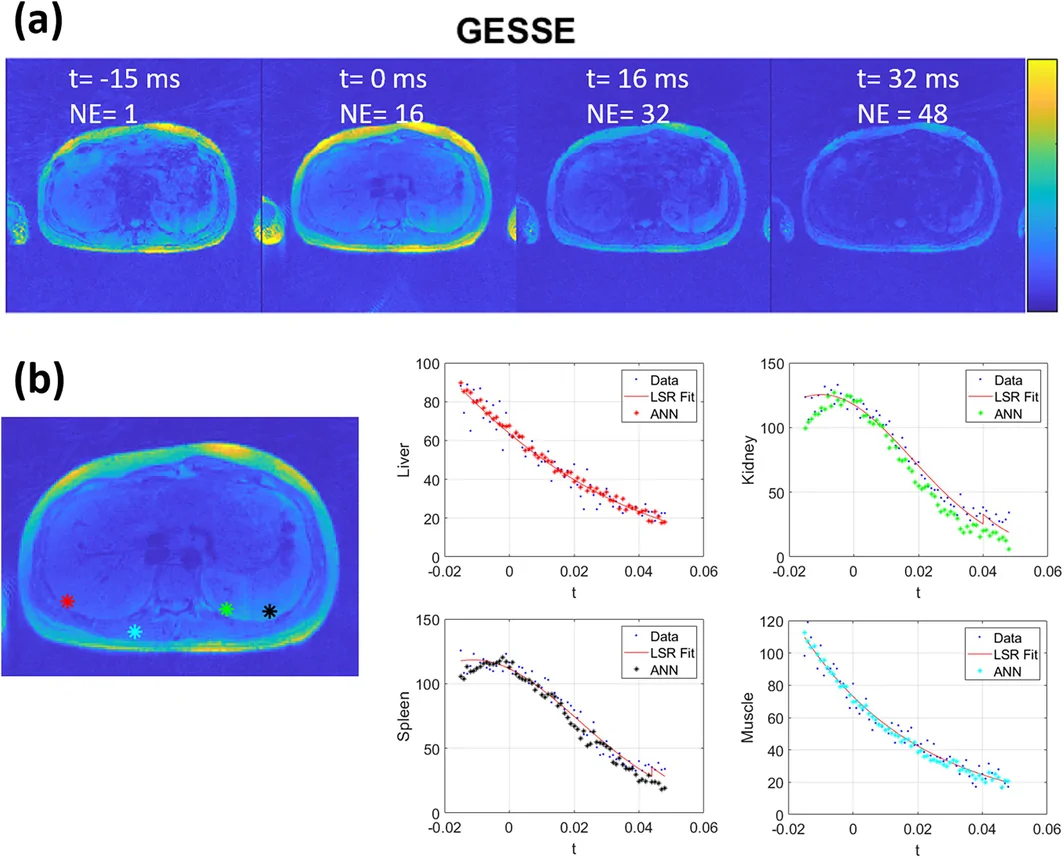
Representative data and model fitting curves. (a) Typical GESSE images at four different time points. (b) Comparison of the LSR and the ANN fit in four typical voxels (liver in red, kidney in green, spleen in black and muscle in cyan). NE means the number of the echo.

Figure 3 displays representative parametric maps of OEF, DBV, and *R*_2_ from a healthy volunteer. The ANN‐based quantification generated high‐quality, anatomically coherent maps with minimal noise amplification. OEF maps demonstrated relatively homogeneous oxygen extraction within the liver parenchyma, while *λ* and *R*_2_ maps revealed organ‐specific variations consistent with known vascular architecture.

**FIGURE 3:**
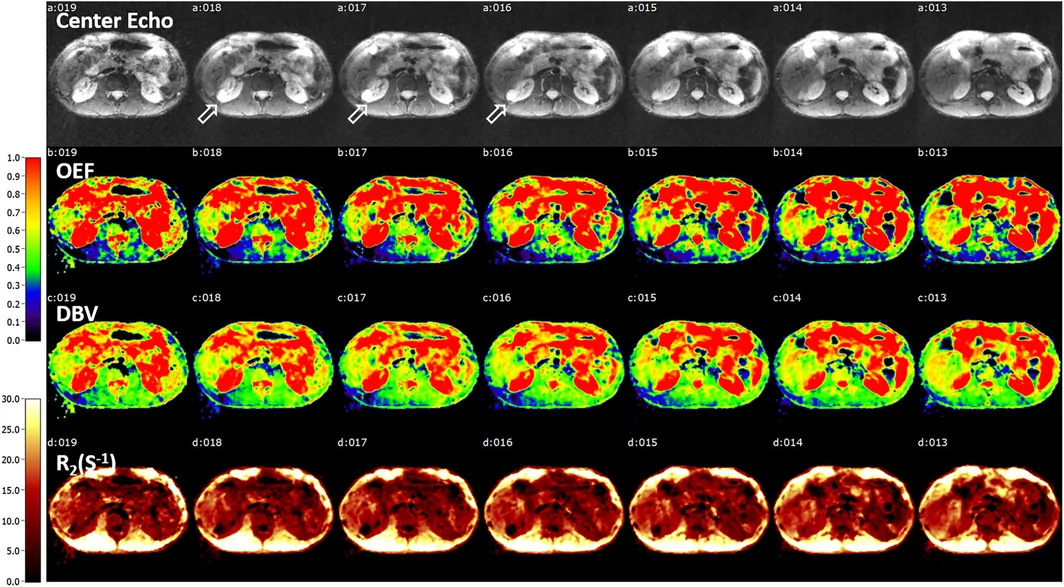
Maps of rGESSE derived parameters from a representative volunteer with a kidney cyst (arrows). (a) Center echo images (TE = 43 ms) as well as (b) OEF, (c) DBV and (d) *R*_2_ maps are presented.

Figure 4 summarizes the quantitative parameters derived across all volunteers. Mean values and inter‐subject variability of OEF, *λ*, and *R*_2_ were extracted from manually placed regions of interest (ROIs) in the liver, kidneys, spleen, and muscle. Across subjects, liver OEF values were within expected physiological ranges (approximately 0.5), and DBV values were highest in the spleen, reflecting its dense vasculature. *R*_2_ values exhibited modest regional variability, consistent with differences in local tissue composition and susceptibility. The corresponding *T*_2_ (1/*R*_2_) values for the liver, kidney, spleen, and muscle were approximately 55, 83, 77, and 46 ms, respectively. Statistically significant differences were observed between all tissue types (*p* < 0.001), except between the kidney and spleen, where no significant difference was found. While hepatic OEF and DBV values were within expected physiological ranges, some kidney and spleen OEF measurements exceeded 100%. In addition, DBV values in the kidney, spleen, and skeletal muscle were elevated relative to expected physiological values, suggesting potential limitations of the current qBOLD modeling framework when applied to these tissues.

**FIGURE 4:**
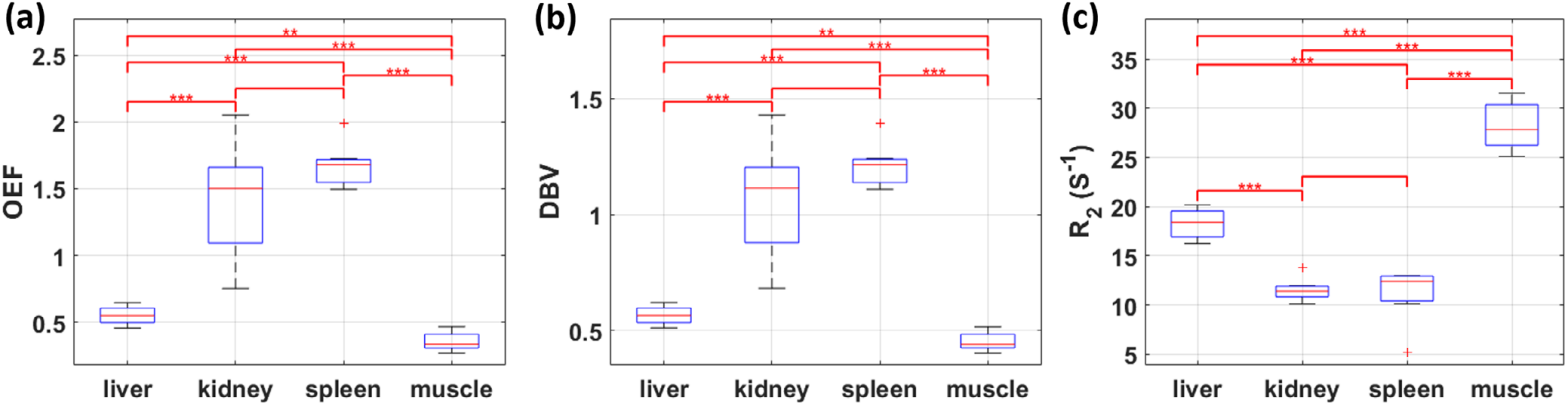
Statistical charts of rGESSE derived parameters: (a) OEF, (b) DBV, and (c) *R*_2_ in different tissues.

The resulting maps from 2D GRE are shown in Figure 5. Compared to the 2D GRE results, rGESSE produced similar estimates of hepatic OEF (0.45 ± 0.03 vs. 0.56 ± 0.01) and DBV (0.51 ± 0.02 vs. 0.56 ± 0.01). However, rGESSE tended to underestimate *R*_2_ in the liver (12.24 ± 0.86 vs. 20.83 ± 1.64). Consistent with theoretical expectations, OEF and DBV values were higher in the spleen and kidney, and our results agree with this prediction.

**FIGURE 5:**
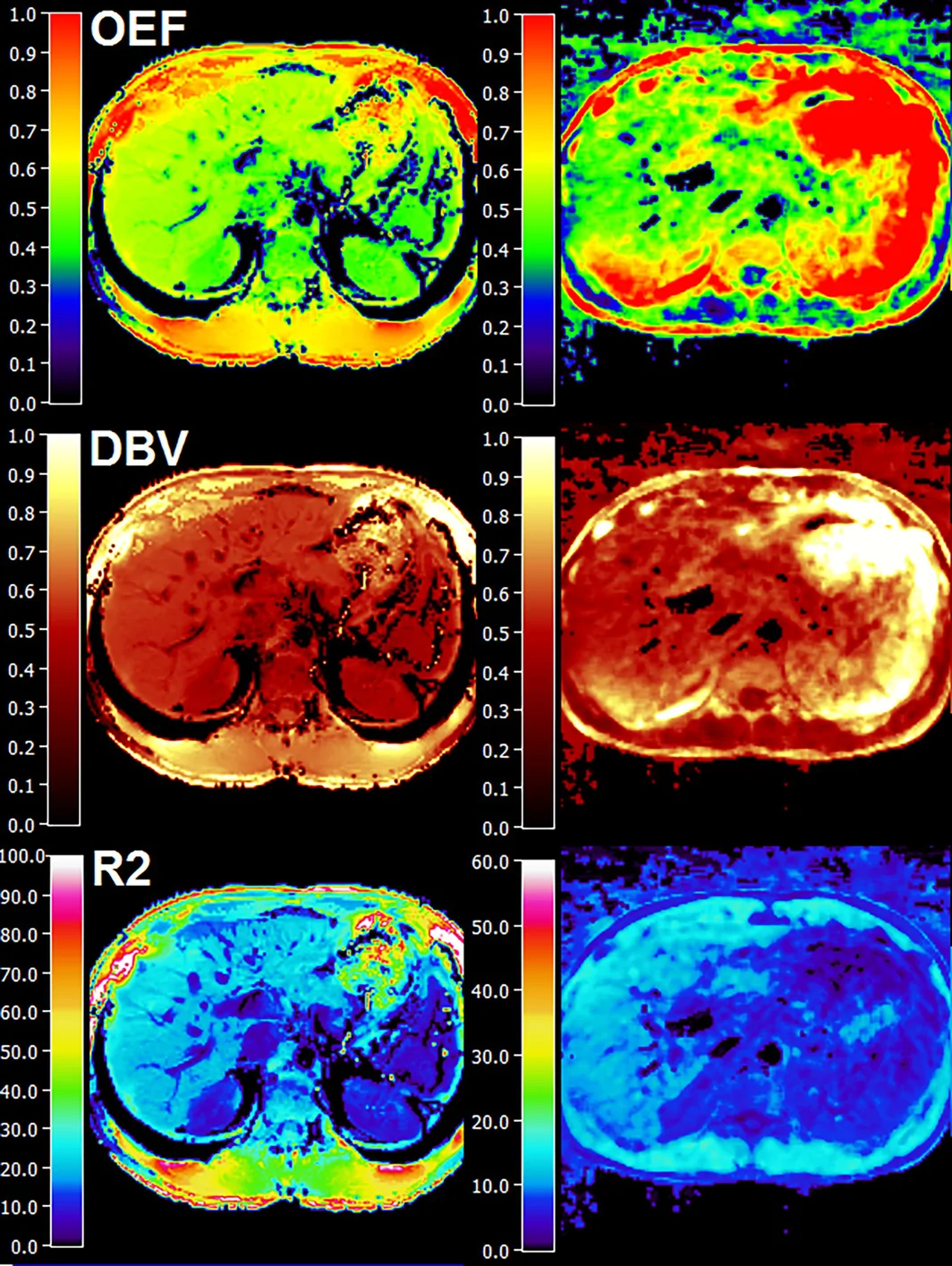
Comparison of single‐slice 2D GRE (left) and rGESSE (right) derided OEF DBV and *R*_2_ maps during free‐breathing. Hepatic OEF (rGESSE: 0.45 ± 0.03 vs. 2DGRE: 0.56 ± 0.01) and DBV (0.51 ± 0.02 vs. 0.56 ± 0.01) are similar, while *R*_2_ (S^−1^) is underestimated in rGESSE (12.24 ± 0.86 vs. 20.83 ± 1.64). Relative contrasts of rGESSE across spleen, kidney, and liver are preserved, indicating physiologically meaningful mapping.

## 4 Discussion

Previous hepatic qBOLD studies were limited to single‐slice, breath‐hold acquisitions with relatively fewer echoes [18], whereas our rGESSE approach enabled multi‐slice free‐breathing acquisition with higher echo sampling density. Overall, this offers more comprehensive spatial coverage and robustness to motion.

The ANN‐based approach offers several advantages over the conventional LSR method. First, the ANN framework enables parameter estimation using the full tissue model, whereas the LSR approach relies on the asymptotic model and is known to fail under realistic clinical imaging conditions [14]. By leveraging the full tissue model, the ANN method utilizes the complete information contained in the GESSE signal, including the region around the spin echo that is discarded in the asymptotic approximation. This more comprehensive use of signal information is expected to yield more accurate and reliable OEF estimates. In addition, the ANN approach is computationally more efficient and does not depend on initialization, avoiding convergence instability commonly encountered with iterative LSR fitting procedures. However, a limitation of this work is that the ANN was trained using signal evolutions generated from the static dephasing qBOLD model. Previous work by Wengler et al. [18] demonstrated significant deviations between hepatic qBOLD signals and Monte Carlo simulations, reflecting violations of the static dephasing regime and simplified vessel geometry assumptions. As a result, training the ANN on an analytical qBOLD model may introduce systematic bias in absolute OEF estimates, particularly in regions with elevated blood volume or complex microvascular organization such as liver. Nevertheless, the model‐based ANN framework enables efficient inversion of a highly nonlinear signal model and provides stable, spatially consistent OEF maps. Future studies will aim to incorporate more realistic forward models, including Monte Carlo‐derived signal simulations and flow‐inclusive formulations, to further improve quantitative accuracy.

In a previous study [18], the mean hepatic venous oxygenation *Y*_v_ in human subjects was reported as 56.8% ± 6.8%, and the mean DBV was 0.190 ± 0.035. In comparison, our study yielded an average hepatic OEF of 55.75% ± 6.88%, which corresponds to a *Y*_v_ of approximately 44.25%, and a hepatic DBV of 0.568 ± 0.039. Assuming the relationship OEF = 1 − *Y*_v_, both OEF and DBV appear to be overestimated in our current implementation. Differences in absolute OEF values between the present study and Wengler et al. may arise from several factors. While *B*_0_ inhomogeneity is expected to contribute to OEF overestimation, particularly in the abdomen, differences in signal modeling may also play an important role. In this work, OEF estimation relies on an ANN trained using a static dephasing qBOLD model, which represents an approximation and may introduce systematic bias, especially in tissues with high blood volume and complex vascular organization such as the liver. In addition, acquisition‐related differences may further contribute to discrepancies across studies. Wengler et al. employed a multi‐echo GRE acquisition, whereas the present work uses an rGESSE‐based sequence, which differs in its sensitivity to macroscopic field gradients, intravoxel dephasing, and refocusing effects. These combined factors are likely to affect the measured signal decay and may partially explain differences in reported OEF values across studies.

The extrahepatic measurements also revealed elevated DBV values in the kidney, spleen, and skeletal muscle, with some estimated OEF values exceeding 100% in the kidney and spleen. These findings indicate that although the proposed method is capable of generating stable parameter maps, the absolute quantification of OEF and DBV outside the liver remains challenging. Overestimation of DBV has previously been reported in qBOLD approaches and has been attributed in part to diffusion effects that violate assumptions of the static dephasing regime underlying the signal model [19]. In abdominal organs, additional factors such as heterogeneous vascular architecture, broad vessel size distributions, susceptibility sources, respiratory motion, and partial‐volume effects may further contribute to model mismatch.

There are several limits of this study. First, rGESSE was a very long measurement that took about 6 min in this implementation. The versatility of combined GE and SE methodology has been demonstrated in various approaches, based on a wide range of sequence implementations, and differing in the readout (single‐ or multiple‐line acquisition), spatial resolution, number and type of echoes used [20]. While EPI outperforms single‐line acquisitions in terms of acquisition time (TA) and temporal resolution, making it the current method of choice for fast imaging sequences, it suffers from distortion, blurring, and local signal loss [21]. Secondly, *B*_0_ field correction was not applied, which may have contributed to artifacts—particularly in the upper lobe region near the lung‐liver interface, where susceptibility‐induced *B*_0_ inhomogeneities are pronounced. These artifacts can degrade signal fidelity and bias parameter estimation in this anatomically challenging area. Future studies should explore the incorporation of *B*_0_ correction techniques, such as voxel spread function (VSF) mapping [22], to mitigate these effects and improve the accuracy of OEF quantification. Finally, this study relies primarily on qualitative assessment and relative inter‐organ comparisons to evaluate the feasibility of free‐breathing hepatic OEF mapping. Although the observed differences between liver, kidney, and spleen are physiologically plausible and support internal consistency, they do not constitute a comprehensive validation of absolute quantitative accuracy. In the absence of an established noninvasive reference standard for hepatic OEF, potential sources of bias, including signal model approximations, residual B_0_ inhomogeneity, respiratory motion, and acquisition‐specific sensitivities remain incompletely characterized. Future work will therefore focus on systematic evaluation of these factors using computer simulations and further theoretical development to better understand and mitigate bias in absolute OEF estimation.

## 5 Conclusion

This study demonstrates the feasibility of using rGESSE for noninvasive, free‐breathing quantification of hepatic OEF. By combining a motion‐robust radial acquisition with ANN‐based signal analysis, we achieved multi‐slice mapping of OEF and related qBOLD parameters across the liver and adjacent abdominal organs. The proposed method produced anatomically coherent parametric maps and demonstrated good sensitivity to regional physiological variation, even under free‐breathing conditions.

## Funding

This work was supported by the Guangdong Science and Technology Program (Grant No. 2025A0505020070) and the SIAT International Joint Lab (Project No. E6G107).

## Conflicts of Interest

The authors declare no conflicts of interest.

## Supporting information


**Figure S1:** Implementation of feedforward ANN. The ANN consisted of a 64‐dimensional input layer, two hidden layers (32 and 10‐dimensional), and a 4‐dimensional output layer.

## Data Availability

The data that support the findings of this study are available from the corresponding author upon reasonable request.
