# Supplementary material for: Free‐Breathing Hepatic Oxygen Extraction Fraction (OEF) Mapping Using Radial GESSE
Source: Magn Reson Med. 2026 Aug 6;96(6):2840–7. doi: 10.1002/mrm.70550 (PMC13619852; doi:10.1002/mrm.70550)
Supplement: Supplementary file 1 [file MRM-96-2840-s001.docx]

**Supplementary**

Figure S1. Implementation of feedforward ANN. The ANN consisted of a 64-dimensional input layer, two hidden layers (32 and 10-dimensional), and a 4-dimensional output layer.
